# Viewing Rural Life through a Public Health Lens

**DOI:** 10.3201/eid3111.AC3111

**Published:** 2025-11

**Authors:** Nkuchia M. M’ikanatha, David P. Welliver, Byron Breedlove

**Affiliations:** Pennsylvania Department of Health, Harrisburg, Pennsylvania, USA (N.M. M’ikanatha); Clarific Services, Rochester, Minnesota, USA (D.P. Welliver); Centers for Disease Control and Prevention (retired), Atlanta, Georgia, USA (B. Breedlove)

**Keywords:** Grandma Moses, Anna Mary Robertson Moses, zoonoses, art–science connection, rural life, soap, sheep

**Figure:**
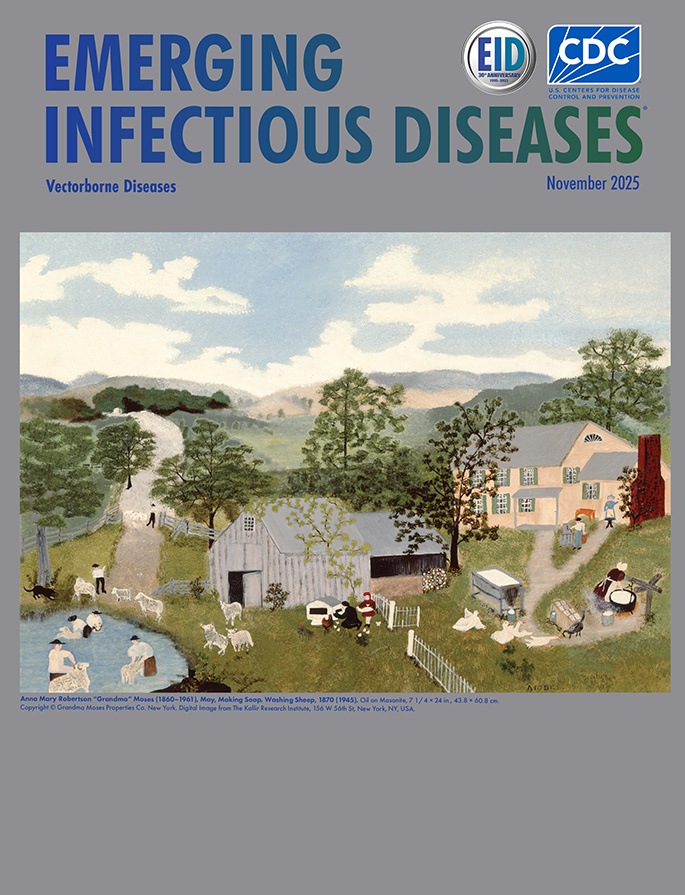
**Anna Mary Robertson (“Grandma”) Moses (1860–1961), *May, Making Soap, Washing Sheep, 1870*** (1945). Oil on Masonite, 17.24 in × 23.94 in × 2 in/43.79 cm × 60.81 cm × 5.08 cm. Copyright © Grandma Moses Properties Co. New York. Digital image from the Kallir Research Institute, 156 W 56th St, New York, NY, USA.

This month’s cover features Grandma Moses’s painting *May, Making Soap, Washing Sheep*, *1870*, a vibrant 1945 depiction of rural life by one of America’s most recognized folk artists. The painting typifies the artist’s extraordinary ability to recall and illustrate the many activities of farm life. Anna Mary Robertson (later fondly known as Grandma Moses) provides a window into the routines of a family farm, where human contact with animals occurred regularly, interactions that can heighten the risk for transmitting zoonotic diseases.

Anna Mary Robertson was born in 1860 in Greenwich in upstate New York and grew up on a farm where she, along with her siblings, contributed to family chores. In her autobiography, *My Life’s History*, Moses recalled a happy childhood with limited schooling. She began working for neighbors for wages at age 12 years. Although she was inclined to painting, Moses lacked the time to develop her artistic talent during her youth and married life.

Anna Mary married Thomas Salmon Moses, an agricultural laborer, in 1887. The couple spent about 2 decades in Virginia’s Shenandoah Valley before settling on a farm in Eagle Bridge, New York, near her birthplace. They named the farm Mount Nebo. Loneliness after her husband’s death in the late 1920s, arthritis (which hindered her ability to stitch embroidery), and her sister’s urging probably motivated Moses, then a septuagenarian, to begin painting.

Tapping into her childhood experiences in a rural setting, Grandma Moses brought numerous authentic details into each of her creations. For example, describing the painting that appears on this month’s cover, she wrote: “Back in the 1870s the farmers would always wash the sheep after a few hot days before shearing, and the wives would make up the year’s supply of hard and soft soap, used on wash day, and [for] house cleaning.” In producing soap on their farm from lanolin extracted from sheep’s wool, Moses and her family continued a practice dating back millennia. Evidence of this enduring method, combining animal fats with wood ash, can be found in various ancient texts from civilizations including Roman, Greek, and Egyptian.

Beginning in her late 70s, until well into her centennial year, Moses created more than 1,500 paintings, a remarkable feat noted by the Smithsonian American Art Museum (Washington, DC). Her success is largely attributed to Otto Kallir, an art dealer who, after escaping Nazi persecution in Austria in 1938, established Galerie St. Etienne (New York, New York). Kallir championed Moses’s work, beginning with a pivotal 1940 exhibition. He meticulously cataloged her paintings and offered crucial insight into her creative process, writing in *Grandma Moses American Primitive*: “The pleasure [she] takes in making a picture, the playful imagination. . . it remains always fresh and fascinating.” The catalog from an exhibit in the National Museum of Women in the Arts (Washington, DC) says that Moses rendered pleasant bucolic scenes and figures that evoked “a world that existed primarily in her imagination.”

Grandma Moses’s nostalgic art depicting “how it used to be” captivated audiences, leading to her widespread recognition beginning in 1940. An early *New York Herald Tribune* report on her October exhibition at Galerie St. Etienne quickly popularized her nickname, “Grandma Moses,” solidifying her public persona. Her work became widely accessible and instantly recognizable to millions of Americans, reproduced on holiday cards, dinner plates, and curtain fabrics. Her appearances on a *Time* magazine cover in 1953 and a 1955 *See it Now* episode hosted by Edward R. Murrow further propelled Grandma Moses into her status as a national icon. Her artwork is held by major institutions, including the Galerie St. Etienne, the Smithsonian American Art Museum, the Metropolitan Museum of Art (New York, New York), and the Bennington Museum (Bennington, Vermont), the last of which maintains the largest public collection of her paintings. In addition, her painting *July Fourth* (1951), a gift to President Harry S. Truman, is part of the White House collection.

In *May, Making Soap, Washing Sheep*, *1870*, set against bucolic landscapes with rolling hills, the depiction of people and animals in a homestead environment mirrors themes explored by the *Emerging Infectious Diseases* journal. The bonneted woman tending to a boiling cauldron evokes a bygone era when family chores included soapmaking and washing sheep before shearing. Nostalgic scenes such as this profoundly resonate, somewhat surprisingly, with topics addressed by *Emerging Infectious Diseases.*

Soap washes away not only dirt and grime but also invisible viruses and bacteria that are on skin. It does not kill microscopic pathogens, but the combination of soap and water helps remove them, thus helping also to reduce illness or infection. “Soap and water and common sense are the best disinfectants,” according to a quotation attributed to Canadian physician William Osler, one of the “Big Four” founding professors of Johns Hopkins Hospital (Baltimore, Maryland) and sometimes referred to as the father of modern medicine.

On the other hand, in some instances, although less frequently, people washing sheep can inhale *Bacillus anthracis*, a spore-forming, drug-resistant bacterium associated with high fatality rates in humans and animals. This bacterium’s spores, however, typically enter the body of workers handling infected sheep, most commonly through carcasses, hides, wool, or contaminated soil, by way of a cut, scratch, or abrasion on the skin. Human contact with sheep, as shown in Grandma Moses’s work, also poses a risk for zoonotic diseases such as Q fever, a highly infectious pathogen.

The presence of domestic fowl, which appear to be ducks or geese, visible near the house introduces an array of other zoonotic risks. For example, Psittacosis (*Chlamydophila psittaci*), an atypical pneumonia, is transmitted to humans by inhaling aerosolized dried droppings from infected birds, an occupational risk highlighted by outbreaks on duck farms. More broadly, the fowl might also harbor parasites, including various tapeworms and protozoa. Furthermore, these birds pose a risk for viral diseases, most notably avian influenza (“bird flu”), such as the circulating H5N1 strain of highly pathogenic avian influenza virus. The presence of that virus is being actively monitored in domestic fowl and livestock across the United States.

In another vignette, chickens gather in front of a gray barn, near a man bent next to a small white structure, with a child in a maroon dress standing nearby. Backyard poultry and their eggs have been associated with outbreaks of emerging drug-resistant strains of *Salmonella infantis*. Other animals present, including a barking dog and horses near the main house on the left, could, without proper precautions, increase the transmission of zoonotic agents such as *Campylobacter* bacteria and *Giardia duodenalis* parasites.

Grandma Moses’s naive style is unquestionably aesthetically pleasing and rewarding. Caution is prudent because the farm landscape in the painting also represents a shared ecosystem for humans, domestic animals (including pets), and microbes. Within this space, known zoonotic pathogens are always present, which underscores the need for integrated One Health surveillance and response.
